# Use and outcomes of amplatz renal dilator for treatment of urethral strictures

**DOI:** 10.1590/S1677-5538.IBJU.2014.0578

**Published:** 2016

**Authors:** Ali Akkoc, Cemil Aydin, Mahir Kartalmıs, Ramazan Topaktas, Selcuk Altin, Yakup Yilmaz

**Affiliations:** 1 Department of Urology, Gazi Yasargil Education and Research Hospital, Diyarbakir, Turkey; 2 Department of Urology, Selahaddin Eyyubi State Hospital, Diyarbakir, Turkey

**Keywords:** Urethral Stricture, Dilatation, Surgical Procedures, Operative

## Abstract

**Introduction:**

Urethral stricture disease is still a major problem in men. Many procedures are available for the treatment of urethral strictures; urethral dilatation is one of the oldest. The blind dilatation of urethral strictures may be a difficult and potentially dangerous procedure. The purpose of this study was to describe safe urethral dilatation using amplatz renal dilator and to report outcomes.

**Materials and Methods:**

From 2010 to 2014, a total of 26 men with primary urethral strictures were managed by urethral dilatation using amplatz renal dilators. The parameters analyzed included presentation of patients, retrograde urethrography (RGU) findings, pre-and postoperative maximum flow rate (Q_max_) on uroflowmetry (UF) and post-void residual urine (PVR). Patients were followed-up at 1.6 and 12 months. The technique described in this paper enables such strictures to be safely dilated after endoscopic placement of a suitable guidewire and stylet over which amplatz renal dilators are introduced.

**Results:**

The mean age of the patients was 57.6 (35–72) years. The median stricture length was 0.82 (0.6–1.5)cm. Pre-operative uroflowmetry showed Q_max_ of 7.00 (4–12) mL/sec and ultrasonography showed PVR of 75.00 (45–195)mL. Postoperatively, Q_max_ improved to 18.00 (15–22)mL/sec (p<0.001) at 1 month, 17.00 (13–21)mL/sec (p<0.001) at 6 months and 15.00 (12–17)mL/sec (p<0.001) at 12 months. The post-operative PVR values were 22.50 (10–60)mL (p<0.001), 30.00 (10–70)mL (p<0.001) and 30.00 (10–70) mL (p<0.001) at 1.6 12 months, respectively. The median procedure time was 15.00 (12–22) minutes. None of the patients had a recurrence during a 12-month period of follow-up.

**Conclusion:**

Urethral dilatation with amplatz renal dilators avoids the risks associated with blind dilatation techniques. This tecnique is a safe, easy, well-tolerated and cost-effective alternative for treatment of urethral strictures.

## INTRODUCTION

Urethral strictures are a common source of referrals to urologists and they are one of the complex issues of urology due to the difficulty of diagnosis, treatment and risk of recurrence (1). The pathology of urethral stricture disease is poorly understood. External trauma generally causes partial or complete disruption of an otherwise normal urethra. How a stricture develops in other circumstances remains unclear but it seems that for whatever reason a scar develops as a consequence of changes in the structure and function of the urethral epithelium and the sub-epithelial spongy tissue causing a fibrotic narrowing of the urethra (2).

Surgical treatment of urethral stricture diseases is rapidly evolving. Currently there are various means of reconstructing the urethra that are almost all comparable in terms of technical easiness, associated morbidity and outcome. However, which one is the best technique has not yet been clearly defined (3). Internal urethrotomy and urethral dilatation are the most commonly performed procedures for urethral stricture disease. The other treatment options include laser urethrotomy, intraluminal stents and urethroplasty (4). The current first-line surgical treatment for urethral strictures includes internal urethrotomy by cold knife and laser (5, 6). However, stricture recurrences and the need for additional surgery are shortcomings of these procedures. Thus, temporary dilatation after internal urethrotomy is also described by some authors for the prevention of stricture recurrence (7, 8). Currently, internal urethrotomy followed by intermittent self-dilatation is the most commonly performed intervention for urethral stricture.

Urethral dilatation has been performed with rigid dilator such as Van Buren and Beniquet dilators or other metal or filiform devices and dilators (9). This modality is used for treating localized and post-urethroplasty urethral strictures. It is performed as a daycare procedure, and should also be considered in patients who are not willing to undergo a reconstructive procedure and/or not fit for anesthesia. Because the traditional dilatation procedure is performed in a blind fashion, potential technical complications at the time of the procedure include excessive bleeding, urethral perforation with extravasation, rectal injury, and false path (9).

In the present study, we describe a safe technique for urethral dilatation in adult populations, report the outcomes of patients and discuss data with related relevant literature.

## MATERIALS AND METHODS

### Patients

This prospective study included 26 men with primary urethral stricture who were operated on during November 2010-September 2014 at Gazi Yasargil Education and Research Hospital in Diyarbakir, Turkey. Inclusion criteria were primary short segment strictures on RGU. Exclusion criteria included patients who had a history of urethral stricture operation, pediatric patients, long strictures (longer than 1.5cm), strictures after distraction injury, and malignant strictures. We have preferred urethroplasty for the strictures longer than 1.5cm and malignant. All patients provided informed consent.

The predominant symptom was a weak urine stream, which occurred in 20 (76.9%) patients. Other symptoms included refractory lower urinary tract infection, urine stream deviation, interrupted urine stream, painful micturition, difficulty initiating urination, paradoxical urinary incontinence and hematuria in two (7.7%), two (7.7%), two (7.7%), two (7.7%), three (11.5%), three (11.5%), and one (3.8%) patient, respectively. None of these patients had any previous history of treatment for stricture. All patients were assessed by whole blood count, BUN, serum creatinine, urinalysis and urine culture. The diagnosis of urethral stricture was based on clinical history, uroflowmetry, ultrasonography, PVR and RGU. RGU was used to locate the site and size of urethral stricture. Ultrasonography was performed to evaluate the upper urinary tract, bladder and PVR.

The etiology of urethral strictures were idiopathic in 3 (11.5%) and iatrogenic in 23 (88.5%) patients. Iatrogenic causes were attributed to transurethral resection of prostate (TURP), transurethral resection of bladder tumor (TURBT), urethral catheterization (UC), retropubic radical prostatectomy (RRP) and open simple prostatectomy (SP) in twelve (52.1%), three (13%), three (13%), three (13%) and two (8.7%) patients, respectively. Regarding stricture location, 2 patients had a stricture at the penile urethra and 1 patient at the bulbous urethra in the idiopathic group; 7 patients had a stricture at the bulbous urethra, 2 patients at the penile urethra and 3 patients at the prostatic urethra in the TURP group; 2 patients had a stricture at the bulbous urethra and 1 patient at the prostatic urethra in the TURBT group; 1 patient had a stricture at the bulbous urethra, 1 patient at the penile urethra and 1 patient at the prostatic urethra in the UC group; 1 patient had a stricture at the bulbous urethra, 1 patient at the membranous urethra and 1 patients at the bladder neck in the RRP group; 1 patient had a stricture at the bulbous urethra and 1 patient at the membranous urethra in the SP group (Table-1). Urine culture yielded>10^5^CFU/mL of susceptible E.Coli in 2 patients. Those patients were treated with suitable antibiotic regimen before the procedure. All patients received first generation cephalosporin preoperatively and it was maintained until removal of urethral catheter. The procedure was performed under spinal anesthesia or sedation in the first 16 (61.5%) patients and local anesthesia (2% intra-urethral lidocaine jelly) in the other 10 (38.5%) patients.

**Table 1:** Urethral stricture characteristics.

		Number of patiens (n)	The percentage of patients (%)
**Patient Symptoms**	Weak urine stream	20	76.9
	Refractory lower urinary tract infection	2	7.7
	Urine stream deviation	2	7.7
	Interrupted urine stream	2	7.7
	Painful micturition	2	7.7
	Difficulty initiating urination	3	11.5
	Paradoxical urinary incontinence	3	11.5
	Hematuria	1	3.8
**Iatrogenic Causes of Structures** *	TURP	12	52.1
	TURBT	3	13
	Urethral catheterization	3	13
	RRP	3	13
	Open simple prostatectomy	2	8.7
**Stricture locations**	Penile urethra	5	19.2
	Bulbous urethra	13	50
	Prostatic urethra	5	19.2
	Membranous urethra	2	7.7
	Bladder neck	1	3.8

* The etiology of urethral strictures were iatrogenic in 23 (88.5%) and idiopathic in 3 (11.5%) patients.

All patients were evaluated with clinical history, urinalysis, urine culture, uroflowmetry, PVR at 1.6 and 12 months, and additional RGU at 6 months postoperatively. The mean follow-up was 12 months. Thewere oriented to come for examination if they have any urinary complaint after 12 months. Surgical success criteria were defined as Q_max_ more than 15mL/sec on UF at 1 month postoperatively and adequate urethral caliber in retrograde urethrography at 6 months. Surgical indications for recurrence during follow-up were of obstructive symptoms and Q_max_ smaller than 10mL/sec (with at least 150cc voiding volume). Patients who needed re-operation due to recurrence of urethral strictures were considered relapse.

### Technique

**Figure 1:**
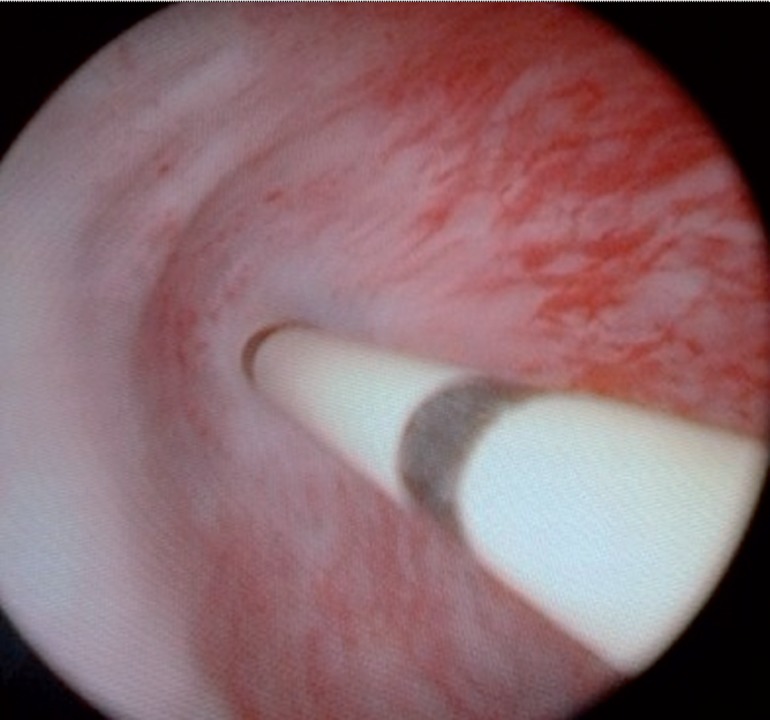
A 5 French (F) open-tip ureteral catheter is manipulated through the stricture and advanced into the bladder.

**Figure 2:**
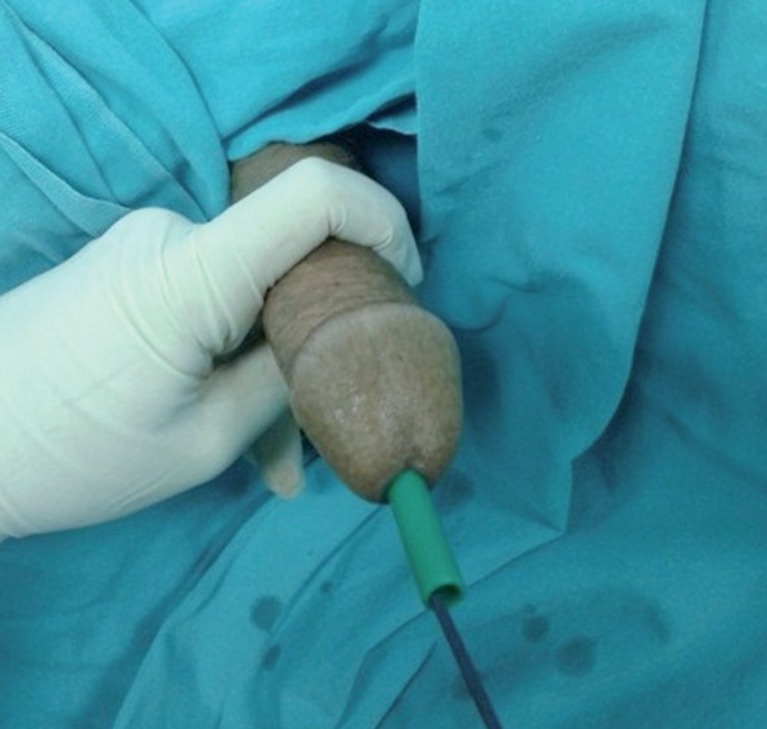
Sequential dilatation is performed with amplatz renal dilators from 10F to 26F by 8F stylet over as in percutaneous renal surgery.

**Figure 3:**
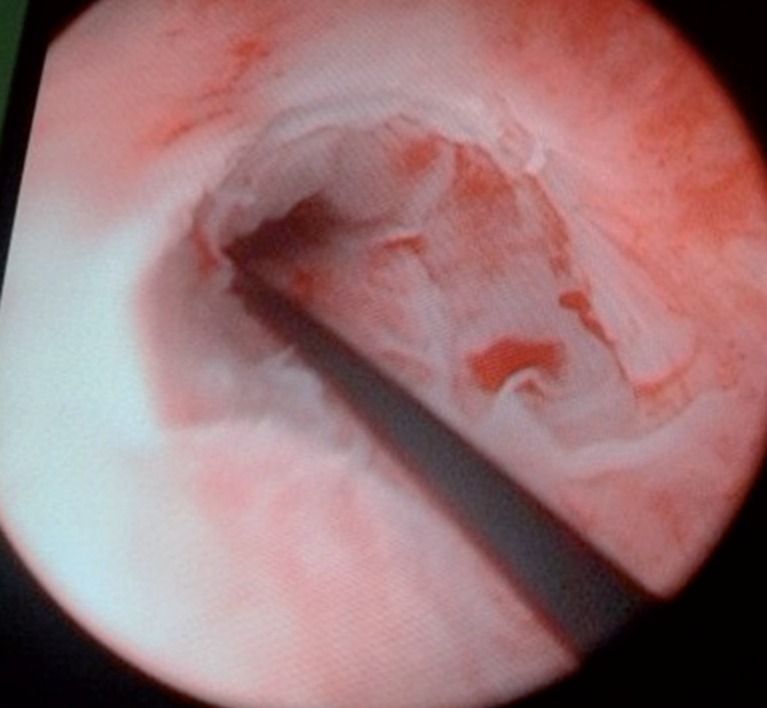
The image of the stricture at the end of the procedure.

Patients are placed in the lithotomy position. Cystoscopy is initially performed to evaluate the urethra and urethral stricture. When the stricture is located, a 5 French (F) open-tip ureteral catheter is manipulated through the stricture and advanced into the bladder (Figure-1). A 0.038 inch stiff hydrophilic guidewire is advanced through the ureteral catheter. Next, the ureteral catheter is removed. We have not encountered any problem in advancing the ureteral catheter. We advise not proceeding even if there is resistance or force needed to advance the ureteral catheter. Sequential dilatation is performed with amplatz renal dilators from 10F to 26F by 8F stylet over as in percutaneous renal surgery (Figure-2). Amplatz dilators are usually safe to use over its 8F stylet and it may be possible to finalize dilatation at different levels. Amplatz dilators are advanced by rotation in direction to the bladder. The appearance of the stricture at the end of the procedure is shown in figure (Figure-3). After the dilatation, a 22F Foley catheter is placed with guidance of the guidewire. We routinely remove the Foley catheter on postoperative day 7.

All statistical evaluations were performed by the Statistical Package for Social Sciences (SPSS) software for Windows, version 15.0 (SPSS Inc., Chicago, IL, USA) Any p value less than 0.05 was considered as significant. The Shapiro–Wilk, Wilcoxon tests were used for analysis of results and expressed as median (minimum-maximum).

## RESULTS

**Figure 4:**
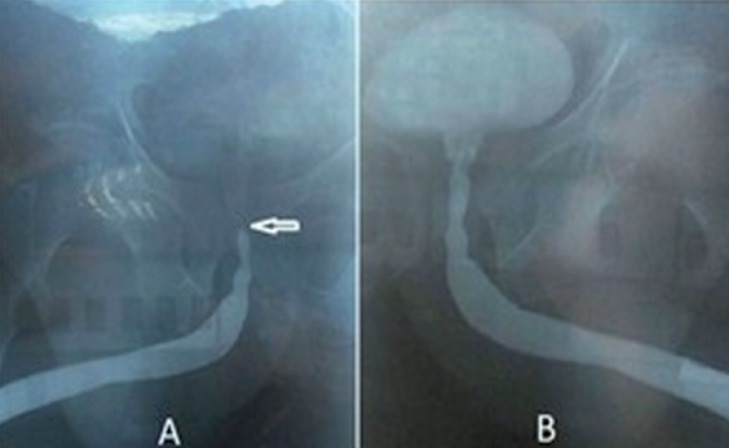
A) Preoperative RGU showing posterior urethral stricture (arrowhead). B) Postoperative RGU showing widely patent urethra after urethral dilatation.

A total of 26 male patients who had short urethral stricture were analyzed. The mean age of patients was 57.6 (35–72) years. The median stricture length was 0.82cm (0.6–1.5). The stricture locations were in the anterior or posterior urethra in 18 (69.2%) and 8 (30.8%) patients, respectively and were single 23 (88.5%) or multiple 3 (11.5%). Preoperative uroflowmetry showed median Q_max_ of 7.00 (4–12)mL/sec and ultrasonography showed median PVR of 75.00 (45–195)mL. Postoperatively, median Q_max_ improved to 18.00 (15–22)mL/sec (p<0.001) at 1 month, 17.00 (13–21)mL/sec (p<0.001) at 6 months and 15.00 (12–17)mL/sec (p<0.001) at 12 months. The postoperative median PVR values were 22.50 (10–60)mL (p<0.001), 30.00 (10–70)mL (p<0.001) and 30.00 (10–70)mL (p<0.001) at 1.6, 12 months, respectively (Table-2). The Q_max_ and PVR values after 1.6 and 12 months were significantly better compared to the preoperative values. The median procedure time was 15.00 (12–22) minutes including cystoscopy and dilatation. The median post-procedure hospital stay was 18.5 (10–24) hours. All procedures were highly accurate and rapid. There were no false path or other significant complications. Three patients had mild hematuria after the procedure. In all patients, adequate urethral caliber was observed in RGU at 6 months postoperatively. The preoperative and postoperative RGU images of a patient with posterior urethral stricture are shown in Figure-4. No complication was observed during the follow-up period. The procedure was found successful in all patients during the 12-month follow-up but 2 (7.7%) patients underwent urethroplasty due to recurrence at 17 and 21 months postoperatively.

**Table 2:** Preoperative and postoperative parameters.

Parameters		Median	Min-Max/Range
**Qmax**	Preoperative	7.00	4–12/8
	1 month*	18.00	15–22/7
	6 months*	17.00	13–21/8
	12 months*	15.00	12–17/5
**PVR**	Preoperative	75.00	45–195/150
	1 month**	22.50	10–60/50
	6 months**	30.00	10–70/60
	12 months**	30.00	10–70/60
Operation time (minute)		15.00	12–22/10

Data are reported as median (min-max/range).

Q_max_: Maximal flow rate (mL/sec); PVR: Post-void residual urine (mL).

* Q_max_; Preoperative versus 1, 6, 12 months postoperative: statistically significant (p<0.001).

** PVR; Preoperative versus 1, 6, 12 months postoperative: statistically significant (p<0.001).

## DISCUSSION

Urethral stricture is one of the most difficult urological problems to cure adequately and it is as old as mankind (10). Many treatment methods have been described according to localization, length of stricture, density of fibrous tissue in the area of stricture, choice of patient or surgeon and experience of surgeon (11). The first option is internal urethrotomy for many cases because of being simple, cost-effective and its repeatability (12). Studies have shown that success rates are 32%-90% and recurrence rates are 38%-75% after internal urethrotomy (11, 13). It has been reported that success rates with dilatation and laser urethrotomy are approximately 60% and 70%, respectively (14, 15). There have been some attempts to establish which surgical method is the most effective and cost–effective in the treatment of male urethral strictures, but clinical data is very limited (16). There is limited randomised, prospective trials comparing the efficacy of dilatation versus internal urethrotomy as initial treatment for urethral strictures. The studies revealed that both methods offer equivalent outcomes, but their effectiveness is reduced with increasing stricture length. Therefore, the authors recommend these methods only for strictures shorter than 2cm and from 2 to 4cm; strictures longer than 4cm should be treated with primary urethroplasty (14). There is no evidence that internal urethrotomy is better than dilatation, but many urologists intuitively believe so (17).

Urethral dilatation has been used as a management technique dating back to the 6th century B.C. (9). They avoid the need for general, spinal, or intravenous anesthesia. It is a simpler, less invasive, a potentially office based procedure that requires lesser degree of surgical expertise and equipment (18, 19). At the present time, blind urethral dilators are used for urethral dilatation widely and complications rates are high. Guidewire-assisted urethral dilatation avoids the risks associated with blind dilatation techniques. Previously, the guidewire-assisted urethral dilatation method had been described with Cosbie Ross and Lister bougies but there was no clear data about the results (20). For a long time, amplatz renal dilators have been used for tract dilatation in percutaneous renal surgery, confidently. But these dilators haven't been used for dilatation of urethral strictures routinelly. Firstly, we had applied this method due to degradation of optical urethrotomy during the operation. Subsequently, we decided that it could be used routinely. Additionally, we think that the technique can be used as an alternative method in conditions where internal urethrotomy is impossible due to technical reasons.

A flow rate less than 10mL/second is more commonly associated with symptoms and with the secondary effects such as recurrent hematuria or recurrent urinary tract infection and with features of overt bladder obstruction on ultrasonography, but this is not necessarily so. If the stricture is troublesome, it should be treated; if not the patient should be kept under review. With a flow rate of less than 5mL/second, abnormalities such as those listed above are much more likely and the patient is potentially at risk of acute retention, although this is a lot less common than one would expect from the severity of the narrowing of the urethra that is seen in such a situation (21). In these patients, treatment is advisable even if symptoms of voiding difficulty are not troublesome. Preoperatively, no patient had acute urinary retention, but the predominant symptom was voiding difficulty in all of our patients.

Temporary dilatation after internal urethrotomy was described by some authors for the prevention of stricture recurrence (7, 8). We have used this technique for these dilatations, too. The procedure time is a little long than blind dilatation but we have felt ourselves more confident than with blind dilatation.

Complications associated with traditional conventional blind dilatation techniques are common, including recurrence with scarring tissue, creation of a false path, impotence, incontinence, and rupture of the rectum and other neighboring organs (22–24). In the review published in 2012, the complications of internal urethrotomy and urethral dilatation were compared and it was observed that intra-operative complications were more frequent in the dilatation group, however the difference was not statistically significant (dilatation 14% versus urethrotomy 11%). Tightness of stricture appeared to be more problematic among the dilatation group (dilatation 8.5% versus urethrotomy 6.7%). For haemorrhage/haematoma, the rate was lower in the dilatation group (dilatation 2.8% versus urethrotomy 3.8%). For false path formation, both dilatation and urethrotomy had almost the same proportion of men experiencing the problem (dilatation 0.94% versus 0.96%). Extravasation and pain seemed to be exclusively associated with the urethrotomy group whilst knotting, breaking and bending of the filiform leader was associated with the dilatation group only (16). These mentioned complications have been minimized with the safe dilatation method we have performed so the surgeon feels more confident. We did not experience any false path and other significant complications in the 26 urethral dilatations we performed. Only three patients had mild hematuria in post procedure period. When the lubricant is used generously, the rigid materials such as amplatz renal dilators provide effective dilatation. These mentioned complications are very rare in a controlled *rotating dilatation.*

The reported duration of catheterization following urethrotomy ranges from 1 day to 3 months. As yet there is no convincing evidence that extending the duration of catheterization has an impact on the outcome (25). Contrary to the popularly held belief, Albers and colleague (26) reported that leaving the urethral catheter in place for 3 days or less is associated with lower recurrence rates (34%), compared to leaving it for 4–7 days or>7 days (recurrence rates of 43% and 65%, respectively). Most studies have reported a catheterization duration of 1–4 days (14, 27). The urethral catheter size does not contribute significantly to the formation of urethral stricture (28). We used 22F Foley catheter and it stayed in the urethra for seven days routinely in all patients. We have thought that using a large-caliber catheter prevents narrowing of scar tissue again in a short period.

Follow-up times after internal urethrotomy (2–96 months) and success rates of internal urethrotomy (8%-100%) range in severe studies (7, 29). Few studies have compared the efficacy of urethral dilatation and internal urethrotomy. In a retrospective study of 199 men with strictures treated at the Mayo Clinic, 101 (67%) underwent dilatation and 39 (26%) underwent direct vision internal urethrotomy. At a median follow-up of 3.5 years, the probability of not requiring re-treatment within 3 years was 65% for dilatation and 68% for urethrotomy, indicating that these procedures were equally efficacious as initial treatment of bulbar strictures (19). Steenkamp, Heyns and coworkers (14, 27) made a prospective randomized trial between dilatation and internal urethrotomy with a group of 100 patients in each treatment. After 4 years, the trend for urethrotomy was better, but statistical significance was not reached.

Most of the refractory strictures appear within the first 12 months (30). In the study by Santucci in 2010, it has been reported that the average time is 9 months for recurrence of urethral stricture after urethrotomy (31). The initial short-term audit reported that all of our patiens had a satisfactory result with respect to the urinary stream and the subsequent radiologic findings in the RGU. Although it has been observed a worsening over time in the Q_max_ parameters of the patients during 12 month follow-up, any recurrence was observed. Only 2 patients (7.7%) had recurrences after the follow-up period (at 17 and 21 months). Urethroplasty was performed in these 2 patients as the second operation.

The results of the present tecnique are better than blind dilatation and it is a potential alternative to internal urethrotomy whencompared with blind dilatation and internal urethrotomy. Additionally, this is a cost-effective procedure. Although cold knife is reusable, it becomes blind after about 10–12 operations. Although percutaneous renal dilatators are disposable, 8–10 operations can be performed by one renal dilator set; and so it reduces costs of the procedures adequately.

We have used this technique in 26 patients over the past 4 years without a technical procedure-related complication. This dilatation procedure may be a safe approach for the patients whose stricture is shorter than 1.5cm, to whom open surgery is not indicated and who doesn't want surgical treatment. However, short follow-up time and limited number of patients are limitations of this study. Further randomized studies are necessary with longer follow-up and comparing it with other treatment procedures of urethral stricture.

## CONCLUSIONS

Guidewire-assisted urethral dilatation with amplatz renal dilators avoids the risks associated with blind dilatation techniques. This technique does not require special material, and it can be performed in any urological operating room. The procedure is safe, practical and cost-effective. Additionally, it can safely be performed under local anesthesia as a day-care procedure. The authors believe that this technique may be a good alternative to internal urethrotomy and other dilatation techniques for treatment of urethral strictures.
